# Repair of segmental radial defect with autologous bone marrow aspirate and hydroxyapatite in rabbit radius: A clinical and radiographic evaluation

**DOI:** 10.14202/vetworld.2017.752-757

**Published:** 2017-07-07

**Authors:** Kalbaza Ahmed Yassine, Benchohra Mokhtar, Hemida Houari, Amara Karim, Melizi Mohamed

**Affiliations:** 1 Laboratory of Agro-Biotechnology and Nutrition in Semi-Arid Regions, Ibn Khaldoun University of Tiaret, Algeria; 2 Department of Veterinary Sciences, Institute of Agronomic and Veterinary Sciences, BATNA-1 University, Algeria

**Keywords:** autologous bone marrow aspirate, bone regeneration, hydroxyapatite, rabbit, radial defect

## Abstract

**Aim:**

Finding an ideal bone substitute to treat large bone defects, delayed union and nonunions remain a challenge for orthopedic surgeons and researchers. Several studies have been conducted on bone regeneration; each has its own advantages and disadvantages. The aim of this study was to evaluate the effect of a combination of hydroxyapatite (HA) powder with autologous bone marrow (BM) aspirate on the repair of segmental radial defect in a rabbit model.

**Materials and Methods:**

A total of 36 male and adult New Zealand rabbit with a mean weight of 2.25 kg were used in this study. Approximately, 5 mm defect was created in the mid-shaft of the radius to be filled with HA powder in the control group “HA” (n=18) and with a combination of HA powder and autologous BM aspirate in the test group “HA+BM” (n=18). Animals were observed daily for healing by inspection of the surgical site, and six rabbits of each group were sacrificed at 30, 60, and 90 post-operative days to perform a radiographic evaluation of defect site.

**Results:**

Obtained results revealed a better and more rapid bone regeneration in the test group: Since the defect was rapidly and completely filled with mature bone tissue after 90 days.

**Conclusion:**

Based on these findings, we could infer that adding a BM aspirate to HA is responsible of a better regeneration process leading to a complete filling of the defect.

## Introduction

Comminuted fractures of long bones, frequently encountered in veterinary practice, could involve varying amount of bone loss. Treatment of such fracture requires not only proper fixation but also an adequate maintaining of the structural integrity at the fracture site by preserving bone fragments and their blood supply. Autologous bone graft is the “gold standard” method for the management of fractures with bone loss, where a piece of bone is taken from another body site, and transplanted into the defect [1]. Although the successfulness of this method is quite high, it is important to point out its limitations related to its low availability and high risk of donor site morbidity [2,3]. The second choice is allografting, using a tissue harvested from another animal of the same species after processing to reduce antigenicity. However, this treatment results in a decrease of incorporation capacity of the graft with host tissue [1] and includes the risk of immune rejection and pathogen transmission to recipient [4].

Instead of using bone graft, researchers have suggested that application of bone cements could be an alternative solution to induce bone regeneration. An ideal bone substitute should be biocompatible, bioresorbable, osteoinductive, osteoconductive, easy to use and with a similar structure to that of bone. Hydroxyapatite (HA) materials are mostly used because of their excellent biocompatibility, osteoconductive properties, and similarity to the inorganic component of human and animal bones [5]. However, these materials lack osteoinductive properties and could not be used alone for the treatment of large bone defect or nonunions, where osteoinductive properties are essential for bone healing.

Bone marrow (BM) derived mesenchymal cells have been used in several attempts to treat bone defects and in combination with bone substitutes to improve them [6-8]. However, the presence of nonadherent osteogenic cells in the BM [9] and the possible cooperation among BM cells types in tissue repair [10]; suggest that using BM aspirate, instead of expanded and purified mesenchymal stem cells, is preferable for bone cell therapy. Moreover, mesenchymal stem cells must be expanded *in vitro* for several weeks to achieve a high and enough number of cells for transplantation, delaying the treatment and increasing both culture-related contamination risks and therapy costs. In contrast, BM aspiration is usually a simple, safe, clean, and low-cost procedure that allows immediate transplantation of cells into the defect site.

The purpose of this study was to evaluate the effect of a combination of HA powder and autologous BM aspirate on the repair of segmental radial defect in a rabbit model.

## Materials and Methods

### Ethical approval

Surgical and handling procedures were performed after approval by the Ethical Committee of the Institute of Agronomic and Veterinary Sciences of BATNA 1 University – Algeria.

### Animals

A total of 36 clinically healthy, 4-month-old New Zealand male rabbit, with a mean weight of 2.25 kg were used in this study. Before any procedures, animals were acclimatized to approaching and handling for at least 7 days in a light and temperature controlled room. Rabbits were kept in separate cages and provided access to standard rabbit diet and water *ad libitum*.

### Experimental design

Rabbits were divided randomly into two groups (18 animals each): Control group “HA,” treatment group “HA+BM” (HA and autologous BM aspirate). A segmental defect of 5 mm has been created in the mid-shaft of radius. Since this defect is considered by most studies as critical-sized and could not be left without a minimal treatment, we have then proceeded to its filling by HA powder in control group “HA,” and with HA powder and fresh autologous bone morrow in “HA+MO” group. Each treatment was awarded to 18 animals, out of which six animals have been sacrificed on 30^th^, 60^th^, and 90^th^ post-operative days to perform a radiographic evaluation of bone healing.

### Anesthesia and animal preparation

Before surgical procedure and after 12 h fasting, animals were first tranquillized by intramuscular injection of 1 mg/kg of acepromazine (Calmivet^®^). Shaved right forelimb and hip area were prepared for aseptic surgical procedure by application of an iodized polyvidone solution (Dermadine^®^). General anesthesia was then obtained by intramuscular injection of 40 mg/kg of ketamine (Imalgene^®^) and 1 mg/kg of xylazine (Xyla^®^).

### Surgical procedure

**Figure-1:**
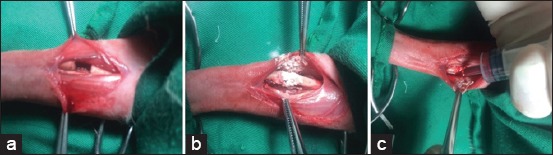
(a) 5 mm bone defect in the mid-shaft of right radius created with a saw-wire. (b) The defect filled with porous hydroxyapatite. (c) Addition of bone marrow aspirate to the defect.

Anesthetized animals were restrained in right lateral recumbency; surgical wraps were applied and a cutaneous incision of 3-4 cm was made on the medial aspect of the right forelimb, approximately equally same distant from the elbow and carpal joints. To expose radius, muscles have been separated using fine blunt scissors. A saw wire was introduced in the interosseous space and used to perform an osteotomy in the mid-shaft of radius. The same procedure was repeated at 5 mm distant from the first osteotomy, and the piece of bone was removed after being detached from its muscular attachment to create a bone defect (Figure-1a). The segmental defect was filled with HA powder (Sigma-Aldrich) (Figure-1b). In the “HA+BM” group, a BM aspiration was performed from iliac crest using an 18 G needle; thus, 0.5 ml of fresh BM aspirate was added to the defect (Figure-1c). After that, muscles have been reapproximated with a running 3/0 polyglactine [9,10] suture, and the skin was closed with simple interrupted 3/0 polyamide suture.

### Post-operative follow-up

Animals were observed daily for healing by inspection of surgical site to detect any swelling, infection or wound dehiscence.

### Radiographic examination

Six animals of each group have been sacrificed at 30, 60, and 90 post-operative days. The right member was detached at the scapulohumeral articulation and two orthogonal radiographs, mediolateral, and anteroposterior have been realized with a numeric radiograph (ENIE Radiologie^®^) at a tension of 65 kV and a charge of 50 mAs. Radiographs have been examined to detect the presence of new bone formation, extent and callus size, bridging of the gap, radiographic density, and remodeling signs.

Radiographic findings were interpreted according to modified Lane and Sandhu, 1987 X-ray scoring systems (Table-1) [11]. Mean scores of each parameter were calculated and scores for different parameters have been additioned to obtain a total radiographic score to quantify the healing in each group. The group with the highest total radiographic score was considered to have better healing.

**Table-1:** Modified Lane and Sandhu [11] radiological scoring system.

Parameters	Score
Reduction in defect size	
Less than 25% reduction	1
25-50% reduction	2
50-75% reduction	3
More than 75% reduction	4
No gap	5
Radiographic density	
No density	0
Slight	1
Moderate	2
Dense	3
Remodeling of bone	
No remodeling	0
Less than 25% reduction in size of callus	1
25-50% reduction in size of callus	2
50-75% reduction in size of callus	3
More than 75% reduction in size of callus and canalization of marrow cavity	4
Maximal possible radiological score	12

### Statistical analysis

After interpretation of radiographs, mean values and standard deviation (SD) were calculated. Data were then analyzed by Student’s t-test (STATISTICA 8), and p˂0.05 was considered statistically significant.

## Results

### Clinical follow-up

No anesthesic death occurred during the experimental procedure; the protocol we have used is secure (all animals used in the study were healthy, so they were considered as ASA 1) and widely recognized because it provides complete surgical anesthesia (tranquillization by acepromazine, analgesia and muscle relaxation by xylazine, and narcosis by ketamine).

In both groups, wound healed by the first intention, and no swelling or apparent signs of infection were detected. Wound dehiscence was observed only in one rabbit from the “HA+BM” group.

### Radiographic examination

Mean±SD values for defect site reduction score, radiographic density, remodeling signs, and total radiographic scores are represented in Table-2.

**Table-2:** Radiographic mean±SD scores of different parameters in groups (“HA” and “HA+BM”), and time periods (30, 60, and 90 days).

Groups	Days	Reduction in defect size	Radiographic density	Remodeling	Total score
HA	30	1.97±0.51*	1.16±0.40**	0.00±0.00**	2.83±0.75*
HA+MO	30	2.33±0.51	2.66±0.51	0.00±0.00	5.00±0.89
HA	60	1.83±0.75*	1.33±0.51**	1.00±0.89*	4.17±1.64*
HA+MO	60	3.83±1.47	2.50±0.54	2.83±1.17	9.17±2.93
HA	90	2.00±1.26**	1.00±1.10*	0.83±1.17**	3.83±3.19**
HA+MO	90	4.16±0.75	2.67±0.51	3.50±0.54	10.33±1.63

* p<0.05,

** p<0.01 between both groups.

SD=Standard deviation, HA=Hydroxyapatite, BM=Bone marrow

**Figure-2:**
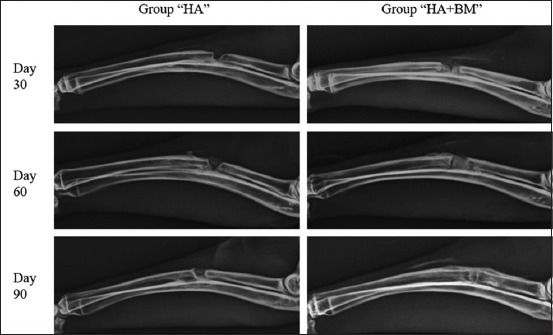
Mediolateral radiographs of animals in “hydroxyapatite (HA)” and “HA+bone marrow (BM)” groups, on day 30, 60, and 90 showing a defect still present in “HA” group and completely filled in “HA+BM” group on day 90.

On day 30, a new bone formation and an important periosteal reaction were observed in “HA+BM” group animals; however, an only mild periosteal reaction was present in “HA” group radiographs. Moreover, defect site reduction score was more important in “HA+BM” group as compared to “HA” group (p˂0.05). Furthermore, radiographic density was also increased in the study group, and radiographic density score in “HA+BM” group was significantly higher than control group (p<0.01). No callus remodeling signs or BM canalization was evident in both groups at 30 days (Figure-2).

Scores for reduction of the defect site have remained unchanged in “HA” group at 60 days, however, a clear reduction of the defect site was observed in group “HA+BM.” Moreover, radiographic density was more important in “HA+BM” group with a highly significant difference as compared to control group (p<0.01). Early remodeling signs were observed at this period in the study group, whereas no remodeling signs were present in control group (Figure-2).

At 90 days, no fracture lines were observed in “HA+BM” group; however, the defect was still present in “HA” group. Radiographic density was significantly higher in “HA+BM” group as compared to “HA” group. In addition, remodeling signs were evident in the study group, and difference between reduction of callus and BM canalization of both groups were highly significant (p<0.01) (Figure-2).

Finally, difference between mean values of total radiographic score of both groups was highly significant throughout the experiment period (p<0.01).

## Discussion

Bone losses, accompanying comminuted fractures or resections of pathological bone lesions, are a major concern in orthopedic surgery because they are often complicated into nonunions if the consolidation process is interrupted by lack of structural integrity or vascularization in the fracture site. Bone healing success mainly depends on the presence of adequate mechanical stabilization and biological competence of the body; pro-osteogenic cells; scoffolds that allow bone growth (osteoconduction), growth factors (osteoinduction); and enough vascularization for an effective nutrient supply [12]. Autologous bone graft, mainly harvested from the iliac crest of the patient and implanted into the defect site, is considered as the “gold standard” for the treatment of nonunions [13]. This technique was the subject of multiple studies with promising results [14,15]. However, the many disadvantages associated with autogenic, allogeneic, and xenogeneic grafts have prompted researchers to find other alternatives to bone grafting. Nowadays, the use of bone cements, proteins, growth factors, and cells is considered as an alternative to transplantation [16].

HA, Ca_10_ (PO_4_)_6_ (OH)_2_, widely accepted as a biocompatible material due to its similarity to the mineral component of bone and teeth [17], has been first introduced clinically in the 1980s [18]. This biomaterial has attracted a large number of researchers and has been widely used as bone filler [19] and for bone tissue engineering scaffolds [5]. The importance of HA materials is primarily due to their excellent biocompatibility, osteoconductive properties, similarity to the inorganic component of bones [5] and their good integration to the defect site [20]. Furthermore, use of HA ceramics avoids many problems associated with autograft, such as morbidity at the harvest site and transmission of infectious diseases, due to ultra-heating process used for preparation.

Despite the many advantages of calcium phosphate ceramics application, several disadvantages can be reported such as the lack of mechanical strength due to their granular structure [21], their poor degradation in physiological environment and finally absence of osteoinductive properties making them unable to induce osteogenic differentiation of stem cells and osteoblasts or to stimulate new bone formation, which is essential for large bone defects regeneration, senile bone regeneration, and tissue engineering [22,23].

With the hypothesis that addition of BM aspirate to HA could have a positive effect on bone formation, we have proceeded to a comparison of bone healing potential in two groups treated by HA powder and its combination with autologous BM aspirate, respectively, after establishing a defect model in the radius of rabbits. This model has previously been reported suitable because there is no need for internal or external fixation, which influences the bone healing process [24].

In our study, inspection of surgical site during first post-operative days have not revealing any swelling or apparent signs of infection. These findings indicate that the material and the technique used for bone filling are biocompatible. In addition, the only case of wound dehiscence detected could be explained by the aggressive behavior of the animal. Ours results are in agreement with those reported in many studies [25-27].

In control group, HA powder has shown good integration to the host bone and promoted a limited new bone formation. This is in agreement with the findings of many studies [28,29] that have used a HA powder to treat radial fractures in humans. However, low radiographic scores were obtained in terms of reducing the bone defect. This could be explained by the slight osteogenic differentiation of stem cells and osteoblasts in the defect site, which led to a poor new bone formation. These findings confirm the lack of osteoinductivity in calcium phosphate biomaterials. Furthermore, the low radiographic density scores registered in control group could be explained by the inability of the few osteoblasts, activated during the slight osteogenic differentiation, to synthesize and mineralize a sufficient bone matrix that could fill the bone defect. Moreover, low scores obtained in terms of remodeling signs in control group can be explained by the absence of osteoclasts, which are the responsible cells of this bone healing phase. These findings confirm the failure of bone consolidation even after a period of 90 days. Our results are in agreement with the general assumption that HA has no osteoinductive activity [30,31] and in defects filled with HA alone, healing process largely depends on the osteoconductive properties of the material. Similar results have been reported by many authors [32-35].

Several studies have been conducted to improve HA ceramics properties. Addition of total blood [36], platelet rich plasma [36,37], bone morphogenetic proteins (BMPs) [16,38] collagen [39], mesenchymal stem cells [40], and BM [41,42] are among the most proposed techniques.

To our knowledge, few studies have used a combination of BM aspirate and HA to treat bone defects; most researchers have focused on the use of mesenchymal stem cells and other growth factors. However, the possible cooperation between the different BM cells in tissue repair [10,43], suggests that use of BM aspirate, instead of mesenchymal stem cells expanded and purified, is preferable for bone defects treatment. Moreover, this technique can bypass the time-consuming and difficult process of mesenchymal stem cells expansion, which must be expanded *in vitro* for several weeks to reach a sufficiently high number of cells for transplantation, which not only delays the treatment but also increases treatment costs and contamination risk related to culture [42].

In our study, a complete bone healing was observed in the group treated by a combination of autologous BM aspirate and HA. The bone defect reduction started precociously; that after 30 days, 25-50% of the defect was already reduced. This new bone formation has continued until the defect was completely filled at 90 days. Moreover, bone density was higher than that observed in control group. These results could be explained by an important osteoblastic activity, which led to an important synthesis and mineralization of a sufficient bone matrix to fill the defect site. Furthermore, early remodeling signs that could be explained by an osteoclastic activity were evident at 60 days and more important at 90 days in this group.

Finally, comparison of total radiographic scores of both groups has revealed a highly significant difference (p<0.01). These findings confirm that addition of BM aspirate to HA has led to an important augmentation of bone healing. This improved bone consolidation is undoubtedly due to the osteogenic potential of BM, reported for the first time by McGraw and Harbin [44] who have used BM as a graft to fill a fibular defect in dogs and have compared it with the defect of the ungrafted contralateral fibulae. Results have indicated that the grafted side with BM was filled with bone as opposed to the other side ungrafted. Indeed, BM contains a mononuclear cell fraction, which contributes to both the vascularization and bone healing cascade. Similarly, BM contains most of growth factors necessary for conversion of mesenchymal stem cells into osteoblasts, which are the first actors of bone healing [45].

Our findings are in agreement with those reported by many authors. In fact, similar results have been reported in a study using autologous BM aspirate in combination with HA granules on tibial sheep’s fractures [31]. Another research has reported comparable findings after using a combination of BM and recombinant human BMP7 [41]. Finally, our results are in concordance with those obtained after using mesenchymal cells loaded on beta-tri-calcium phosphate granules for treating mandibular deficits in rabbits [46].

## Conclusion

The results of our study can, therefore, confirm that addition of BM aspirate improves the characteristics of HA powders and that the recently proposed strategy of combining BM to bone grafts, commonly called “transplants revitalization,” can be considered as a promising option for treatment of fractures with bone losses or nonunions.

## Authors’ Contributions

KAY, AK, and MM planned and design the study and performed the surgical procedure and radiographic examination. BM and HH helped in the clinical follow-up. All authors read and approved the final manuscript.
